# The Effects of Enteral Nutrition on the Intestinal Environment in Patients in a Persistent Vegetative State

**DOI:** 10.3390/foods11040549

**Published:** 2022-02-15

**Authors:** Hiroshi Matsuoka, Takumi Tochio, Ayako Watanabe, Kohei Funasaka, Yoshiki Hirooka, Tenagy Hartanto, Yuka Togashi, Misa Saito, Yuichiro Nishimoto, Yoshinori Mizuguchi, Masanobu Kumon, Chieko Sakuragi, Kouichi Suda, Yuichi Hirose, Isao Morita

**Affiliations:** 1 Department of Surgery, Fujita Health University, Toyoake 470-1192, Aichi, Japan; mats1025@fujita-hu.ac.jp (H.M.); ko-suda@nifty.com (K.S.); 2 Department of Gastroenterology and Hepatology, Fujita Health University, Toyoake 470-1192, Aichi, Japan; ayako.watanabe@fujita-hu.ac.jp (A.W.); k-funa@fujita-hu.ac.jp (K.F.); yoshiki.hirooka@fujita-hu.ac.jp (Y.H.); 3 Metabologenomics, Inc., Tsuruoka 997-0052, Yamagata, Japan; tenagy@metagen.co.jp (T.H.); yuka.mori@metagen.co.jp (Y.T.); misa.saito@metagen.co.jp (M.S.); yuichiro.nishimoto@metagen.co.jp (Y.N.); mizuguchi@metagen.co.jp (Y.M.); 4 Department of Neurosurgery, Fujita Health University, Toyoake 470-1192, Aichi, Japan; d.m.kumon@gmail.com (M.K.); yhirose@fujita-hu.ac.jp (Y.H.); iokuma@fujita-hu.ac.jp (I.M.); 5 Nursing Department, Fujita Health University Hospital, Toyoake 470-1192, Aichi, Japan; luna0929@fujita-hu.ac.jp

**Keywords:** enteral nutrition, intestinal environment, dysbiosis, probiotics

## Abstract

Enteral nutrition (EN) is a rational approach to providing nutritional intake via the intestines in patients who are unable to tolerate parenteral nutrition. We conducted a preliminary study to investigate the effects of EN on the intestinal environment in 10 patients in a persistent vegetative state (PVS) (*n* = 5 each in the EN and EN with probiotics; *Clostridium butyricum MIYAIRI 588*) groups compared with 10 healthy controls. The results of 16S amplicon sequencing of the intestinal microbiota showed that EN led to dysbiosis with a decrease in α-diversity and an obvious change in β-diversity. A particularly significant decrease was seen in useful intestinal bacteria such as *Bifidobacterium* and butyrate-producing bacteria. Analysis of intestinal metabolites also supported these results, showing significant decreases in butyric and pyruvic acid after EN. Although *C. butyricum* *MIYAIRI 588* improved some intestinal metabolites that were decreased after EN, it did not improve the dysbiosis of the intestinal microbiota. These findings indicate that EN causes dysbiosis of the intestinal microbiota and an imbalance in some intestinal metabolites in patients in a PVS. Moreover, although *C. butyricum* *MIYAIRI 588* improved the imbalance of some intestinal metabolites after EN, it did not prevent dysbiosis of the intestinal microbiota.

## 1. Introduction

Enteral nutrition (EN) is a method of administering carbohydrates, proteins, lipids, electrolytes, vitamins, and trace elements enterally, and is widely used in various fields of perioperative nutrition because it allows for high-energy administration and utilizes the body’s digestive and absorptive capacity. On the other hand, the effects of EN on the body outside of nutritional supplementation have not been fully elucidated.

Recent studies have reported that changes in the intestinal environment, including the intestinal microbiota and metabolites, are associated with various diseases, including metabolic diseases, allergies, cancer, and neurological disorders. For example, Koh et al. [1] reported that dysbiosis of the intestinal microbiota, characterized by an increase of harmful bacteria such as *Eggerthella* and *Streptococcus*, induces the production of imidazole propionate, which is one of the factors in the pathogenesis of diabetes. Feehley et al. [2] reported that a decrease in *Anaerostipes caccae,* an intestinal bacterium that produces butyrate, is associated with food allergies. Nishikawa et al. [3] reported that intestinal dysbiosis is involved in Parkinson’s disease.

Although EN is properly designed to maintain the organism from a nutritional point of view, it is not designed to be properly utilized by intestinal microbiota. We speculated that EN induces dysbiosis of the intestinal microbiota and is closely related to the onset and progression of various diseases via the deterioration of the intestinal environment. On the other hand, few studies have been conducted to investigate this hypothesis.

A persistent vegetative state (PVS) refers to cognitive impairments caused by brain injury, as typified by traumatic brain injury or cerebral hemorrhage, and is one of the medical conditions for which EN is widely used. In patients in a PVS, EN is often used for a long period of time at home after the acute phase, and during this process, some patients develop digestive disorders such as paralytic intestinal obstruction, infections such as pneumonia, or metabolic disorders such as diabetes [4,5].

Given this background, we aimed to elucidate the effects of EN on various diseases by investigating the intestinal environment in patients in a PVS after the acute phase. As a first step, we performed the present preliminary study to investigate the effects of EN on the intestinal environment in 10 patients in a PVS by comparing them with 10 healthy controls.

## 2. Materials and Methods

### 2.1. Clinical Study

The present study on patients in a PVS was reviewed by the Ethics Committee of Fujita Medical University (HM20-044; UMIN: 000045641). The study on healthy controls was reviewed by the Ethics Committee of Chiyoda Paramedical Care Clinic (MTG18C3; UMIN: 000035548). Fecal samples were collected from 10 patients in a PVS who had been receiving EN for more than 60 days (*n* = 5 each in the EN and EN with probiotics (EN pro (+)) groups; 7 males, 3 females; median age 62.6 years; age range 35–79 years) and 10 healthy controls (healthy (HT) group: 5 males, 5 females; median age 37.5 years, age range, 30.3–48.8 years) using a collection container (Sarstedt K.K., Numbrecht, Germany)). RACOL-NF Liquid (Otsuka Pharmaceutical Co., Ltd., Tokyo, Japan) was used as the EN for all patients. *Clostridium butyricum MIYAIRI 588* (MIYA BM; MIYARISAN Pharmaceutical Co., Ltd., Tokyo, Japan) was used as the probiotic (1 × 10^7^ cfu/g, dose: 3 g/day). The exclusion criteria were as follows: use of antibiotics for 1 week or more within the prior 6 months, use of gastric or bowel medication such as proton pump inhibitors within the prior 2 weeks, and use of probiotics other than *C. butyricum MIYAIRI 588* within the prior 1 month. Collected fecal samples were used for 16S rRNA amplicon sequencing and metabolomic analysis.

### 2.2. 16S Amplicon Sequencing Analysis of Intestinal Microbiota

Fecal samples were initially lyophilized using a VD-800R lyophilizer (TAITEC Co., Ltd., Saitama, Japan) for at least 18 h. Freeze-dried feces were disrupted with 3.0 mm zirconia beads by vigorous shaking (1500 rpm for 10 min) using Shake Master (Biomedical Science Co., Ltd., Tokyo, Japan). Fecal samples (10 mg) were suspended with DNA extraction buffer containing 400 μL of 10% (*w*/*v*) SDS/TE (10 mM Tris-HCl, 1 mM EDTA; pH 8.0) solution and 400 μL of phenol/chloroform/isoamyl alcohol (25:24:1). Feces in the mixture buffer were further disrupted with 0.1 mm zirconia/silica beads by vigorous shaking (1500 rpm for 5 min) using Shake Master. After centrifugation at 17,800× *g* for 5 min at room temperature, DNA extraction from fecal samples was performed using an automated DNA extraction machine (GENE PREP STAR PI-480; Kurabo Industries, Ltd., Osaka, Japan) according to the manufacturer’s protocol. After extraction, the V1–V2 region of the 16S rRNA gene was amplified using primers [6]. 27F-mod (AGRGTTTGATYMTGGCTCAG) and 338R (TGCTGCCTCCCGTAGGAGT). The amplified DNA was sequenced using MiSeq (Illumina, San Diego, CA, USA), according to the manufacturer’s protocol.

### 2.3. Metabolomic Analysis of Intestinal Metabolites by LC-TOFMS

#### 2.3.1. Chemicals and Reagents

Camphor-10-sulfonic acid (CSA) for internal standard (IS), 2-hydroxyisobutyric acid, malonic acid, succinic acid, lithium L-lactate (lactic acid), cholic acid (CA), taurocholic acid (TCA), glycocholic acid (GCA), taurochenodeoxycholic acid (TCDCA), taurodeoxycholic acid (TDCA), taurolithocholic acid (TLCA), and lithocholic acid (LCA) were purchased from Sigma-Aldrich (St. Louis, MO, USA). Acetic acid, isovaleric acid, valeric acid, sodium butyrate (butyric acid), chenodeoxycholic acid (CDCA), liquid chromatography–mass spectrometry (LCMS)-grade methanol, and isopropanol were purchased from Wako Pure Chemical Industries, Ltd. (Osaka, Japan). Propionic acid and isobutyric acid were purchased from Nacalai Tesque (Kyoto, Japan). Pyruvic acid, 2-methylvaleric acid, 4-methylvaleric acid, caproic acid, tauroursodeoxycholic acid (TUDCA), glycoursodeoxycholic acid (GUDCA), ursodeoxycholic acid (UDCA), glycochenodeoxycholic acid (GCDCA), glycodeoxycholic acid (GDCA), and deoxycholic acid (DCA) were purchased from Tokyo Chemical Industry Co., Ltd. (Tokyo, Japan). LCMS-grade H_2_O was purchased from Kanto Chemical Co., Inc., (Tokyo, Japan). Reagents for short-chain fatty acid (SCFA) derivatization were obtained from YMC Co., Ltd. (Kyoto, Japan). Formic acid and ammonium formate for mobile phases were purchased from Fisher Chemical (Newington, NH, USA) and Honeywell Fluka (Seelze, Germany), respectively.

#### 2.3.2. Fecal Sample Pretreatment and Standard Preparation

Fecal samples were lyophilized and crushed into powder as previously described. Approximately 10 mg (±0.5 mg) of fecal samples was suspended in 300 μL of IS solution (20 μM CSA in 50% H_2_O/methanol). These fecal suspensions were then further disrupted with 0.1 mm zirconia/silica beads by vigorous shaking (1500 rpm for 10 min) using Shake Master neo and centrifuged at 20,400× *g* for 5 min. The supernatants were then transferred into micro centrifugal filters (Ultrafree-MC, GV 0.22 μm; Merck, Kenilworth, NJ, USA) that were previously washed with 200 μL LCMS-grade H_2_O and 200 μL LCMS-grade methanol by centrifugation at 1000× *g* for 3 min followed by aspiration. The supernatants transferred to the micro centrifugal filter were then centrifuged at 9100× *g* for 5 min. The filter parts were removed, and the filtered samples were stored at –80 °C until measurement. For bile acid (BA), 80 μL filtered stool samples can be directly transferred into vials and measured by LC-TOFMS. For SCFA, the filtered samples were first diluted 100× in IS solution and derivatized according to the manufacturer’s instructions (YMC Co., Ltd., Kyoto, Japan).

Standard solution stocks were prepared by mixing each of the 14 types of BA and 14 types of SCFA at a final concentration of 200 μM in 50% H_2_O/MeOH. For the standard curve, the mixed standard solution stock was further diluted in IS solution by serial dilution. Standard solution for BA was prepared from 2 nM to 100 μM, while standard solution for SCFA was prepared at higher concentrations (20 nM to 1 mM) and derivatized similarly to pretreatment of fecal sample SCFA.

#### 2.3.3. LC-TOFMS Condition

An LC instruments set (Agilent Technologies 1260 Infinity II, Santa Clara, CA, USA) was connected to a time-of-flight mass spectrometer (TOFMS; Agilent Technologies G6230B) with an electrospray ionization source (Dual Agilent Jet Stream ESI). TOFMS detection was operated in negative ionization mode using the following scan source parameters: capillary voltage 3.5 kV, ion source gas temperature 300 °C, drying gas flow 10 L/min, and sheath gas temperature 350 °C with sheath gas flow 11 L/min. The column used was ZORBAX Extend-C18 (2.1 × 50 mm, particle size 1.8 μm; Agilent Technologies, P/N: 727700-902), and the column temperature was set at 45 °C. Column equilibration was carried out 1 day before actual measurement by passing mobile phases A and B at the starting ratio according to each measurement method.

#### 2.3.4. LC-TOFMS Measurement

Mobile phases A and B were used at a flow rate of 0.3 mL/min with a gradient system. For SCFA measurement, the gradient was started at 90% mobile phase A (0.1% formic acid/H_2_O) and 10% mobile phase B (0.1% formic acid/methanol), and then gradually changed from 70% mobile phase A and 30% mobile phase B at 0.1 min to 40% mobile phase A and 60% mobile phase B for 2 min, 10% mobile phase A and 90% mobile phase B for 6 min, and finally to 10% mobile phase A and 90% mobile phase B for washing. For BA measurement, the gradient was started at 90% mobile phase A (5 mM ammonium formate in H_2_O) and 10% mobile phase B (5 mM ammonium formate in methanol), and then changed from 45% mobile phase A and 55% mobile phase B at 0.1 min to 100% mobile phase B for 9 min, and finally washed with 100% mobile phase B for 10 min. The injection volume was 1 μL per sample, and the sample temperature was kept at 4 °C during measurement. To avoid carry-over from previous samples, the injection path was cleaned by multi-washing with 100% isopropanol followed by 100% H_2_O. The measurement was controlled by Agilent MassHunter Workstation Data Acquisition software (Version B.08.00, Build 8.00.8058.3 SP1, Santa Clara, CA, USA), and the obtained data were analyzed using Agilent MassHunter Quantitative Analysis (for TOF) software (Version B.08.00, Build 8.0.598.0).

### 2.4. Analysis of Bioinformatics and Statistical Analysis

QIIME2 (version 2019.10) was used for 16S rRNA gene analysis [7]. The primer base was trimmed by cutadapt (options: –p-discard-untrimmed) [8]. Sequence data were processed using the DADA2 pipeline for quality filtering and denoising (options: –p-trunc-len-f 230 –p-trunc-len-r 130). The filtered output sequences were assigned to taxa using the “qiime feature-classifier classify-sklearn” command with the default parameters [9]. Silva SSU Ref Nr 99 (version 132) was used as a reference database for taxonomy assignment [10]. The Shannon index was calculated using the “qiime diversity alpha-rarefaction” command with the default parameters. Principal component analysis (PCA) plots, which visualize the beta-diversity, were constructed using SPSS Statistics version 26.0 (IBM). Differences in overall intestinal microbiota structure among the HT, EN, and EN pro (+) groups was assessed using the permutational multivariate analysis of variance (PERMANOVA) test for Euclidean distance as implemented in scikit-bio (version 0.5.5; all default parameter). Statistical analysis of the Shannon index, the occupancy of each intestinal microbiota (Medium IQC 25–75%), and the amounts of metabolites was performed by Mann–Whitney *U* tests using SPSS Statistics (IBM), and differences at *p* < 0.05 were considered significant.

## 3. Results

### 3.1. Analysis of Intestinal Microbiota

**Figure 1:**
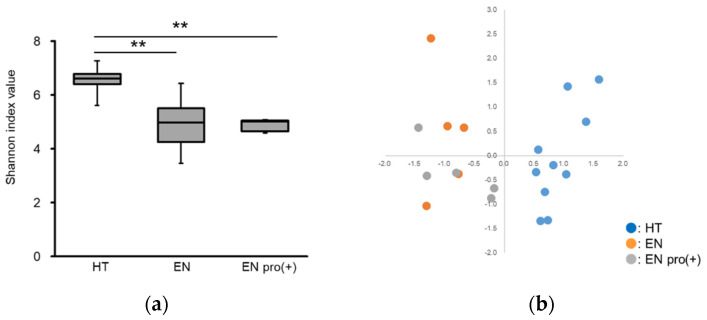
The α-diversity and β-diversity data of the intestinal microbiota in each experimental group at the genus level. (**a**) Shannon index value. (**b**) Two-dimensional PCA analysis. A plot of the first primary component (PC1) on the horizontal axis and the second primary component (PC2) on the vertical axis; each plot represents the microbiota structure of individual samples. Plots are differently colored as follows: HT, blue; EN, orange; EN pro (+), gray. ** significant difference (*p* < 0.01) compared with HT.

The Shannon index value of the intestinal microbiota in the EN group was significantly lower than that in the HT group. On the other hand, no significant difference in the index value was found between the EN and EN pro (+) groups (Figure 1a). The analysis of beta-diversity in the intestinal microbiota indicated that there was significant differences among the HT, EN, and EN pro (+) groups (PERMANOVA, *p* = 0.003, R^2^ value = 0.308). The results of a comparison of the occupancy rate of each intestinal bacterium between groups indicated that intestinal bacteria with a high occupancy rate in the HT group, such as *Blautia*, *Faecalibacterium*, *Bifidobacterium*, *Fusicatenibacter*, *Anaerostipes*, and *Roseburia*, were significantly decreased in the EN and EN pro (+) groups at the genus level. On the other hand, *Parabacteroides* were significantly increased in the EN compared with the HT and EN pro (+) groups (Table 1).

### 3.2. Analysis of Intestinal Metabolites

The results of the metabolomic analysis of intestinal metabolites showed that butyric acid, pyruvic acid, and TCDCA were significantly lower and 2-hydroxyisobutyric acid was significantly higher in the EN than in the HT and EN pro (+) groups (Table 2).

## 4. Discussion

In the present preliminary study, we investigated whether various diseases that occur during EN among patients in a PVS may be related to the intestinal environment. The results showed that EN remarkably decreased the diversity and altered the composition of the intestinal microbiota (Figure 1a,b), and in particular, caused a significant decrease in the occupancy of useful intestinal bacteria such as *Blautia*, *Faecalibacterium*, *Bifidobacterium*, *Anaerostipes*, and *Roseburia*, which were highly prevalent in the HT group (Table 1).

The results of heat map also indicated that intestinal microbiota in PVS patients with EN were an obvious dysbiosis compared to the HT group (Figure S1).

*Faecalibacterium*, *Anaerostipes*, and *Roseburia* are butyrate-producing bacteria that produce butyric acid, a type of SCFA involved in improvement in lifestyle-related diseases and allergies [11,12,13,14].

*Bifidobacterium* produces acetic acid and lactic acid and inhibits infection with pathogenic bacteria [15]. These results suggest that EN may increase the risk of immune abnormalities, metabolic diseases, and infections resulting from a decrease in useful intestinal bacteria such as *Bifidobacterium* and butyrate-producing bacteria in patients in a PVS. In fact, the use of long-term EN has increased the prevalence of various diseases, such as infectious and digestive disorders [4,5]. However, clinical studies are needed to confirm the relationship between dysbiosis of the intestinal microbiota and the risks involved with the use of long-term EN. On the other hand, the effects of *Parabacteroides*, which increased after EN, on the body remain poorly understood. In the future, the relationship between *Parabacteroides* and EN should be clarified in bacterial transplantation studies.

In addition, the results of the intestinal metabolite analysis revealed that EN caused significant decreases in pyruvic acid, butyric acid, and TCDCA, a type of primary bile acid secreted by the liver, and a significant increase in 2-hydroxyisobutyric acid.

Pyruvate is a typical component of intestinal metabolites produced from glucose by many intestinal bacteria and has been reported to activate intestinal immunity in the small intestine [16]. Butyric acid is produced by butyrate-producing bacteria from SCFAs such as acetic acid and is involved in the suppression of inflammation and improvement of metabolism [17]. As we examined the relationship between intestinal microbiota and metabolites by Spearman correlation analysis, the results showed that butyric acid and pyruvic acid were a significantly positive correlation with butyrate-producing bacteria such as *Anaerostipes* and *Roseburia* (Table S1).

This result supports that of 16S amplicon sequencing analysis of the intestinal microbiota, demonstrating that EN causes decreases in pyruvate and butyrate following a decrease in intestinal microbiota diversity and a marked decrease in butyrate-producing bacteria.

On the other hand, the present study is a preliminarily study, and it will be necessary to collect data from large-scale clinical trials in the future.

The effects of intestinal microbiota on the decrease in TCDCA and the increase in 2-hydroxyisobutyric acid were unclear.

The effects of these components on the intestinal microbiota will need to be investigated in a future study.

Schneider et al. [18] compared the intestinal environment between healthy subjects and patients receiving EN, reporting that EN led to decreases in *Bifidobacteria*, anaerobic bacteria, and butyric acid, but no changes in *Clostridium*. Their results are very similar to and support the validity of our findings.

*C. butyricum MIYAIRI 588* is a probiotic with intestinal action that produces SCFAs such as acetic acid and butyric acid [19,20]. The combined use of *C. butyricum MIYAIRI 588* and EN improved the intestinal metabolites, which were increased or decreased by EN, but not the dysbiosis of the intestinal microbiota. Improved intestinal metabolites may have been produced by the administered *C. butyricum MIYAIRI 588*. These results suggest that *C. butyricum MIYAIRI 588* may not be an essential means for resolving the adverse effects of EN on the intestinal environment. We speculate that the combination of prebiotics, which selectively increase useful intestinal bacteria [21], may be effective for reconstituting specific intestinal bacteria populations disrupted by EN. To prove this hypothesis, we plan to conduct clinical trials using prebiotics to increase selectively the intestinal bacteria reduced by EN. On the other hand, because we examined only *C. butyricum MIYAIRI 588*, it is possible that the use of other probiotics may improve the reconstitution of the intestinal microbiota; therefore, the effects of other probiotics should be investigated.

On the basis of these results, we concluded that EN adversely affects the intestinal environment owing to dysbiosis of the intestinal microbiota and the imbalance of several important intestinal metabolites in the 10 patients in a PVS. Moreover, the combined use of *C. butyricum MIYAIRI 588* appears to improve the imbalance of intestinal metabolites after EN, but not dysbiosis of the intestinal microbiota.

On the other hand, this preliminary study still has the following issues: there are differences in the age groups of healthy subjects and PVS patients, and multiple probiotics and prebiotics were not examined. In the future, it will be essential to conduct large-scale clinical studies and some studies combining multiple probiotics and prebiotics. By conducting these studies, we speculate that in the future, we may be able to solve various diseases associated with the use of EN.

## Figures and Tables

**Table 1:** Types of intestinal bacteria at the genus level (intestinal bacteria % by group).

Intestinal Bacteria (Genus)	HT	EN	EN Pro (+)
*Bacteroides*	15.66 (11.35–20.67)	14.07 (8.09–41.49)	31.68 (4.20–37.73)
*Blautia*	10.95 (8.99–12.32)	0.31 (0.00–1.76) **	1.52 (0.00–4.30) **
*Faecalibacterium*	10.37 (3.89–14.95)	0.00 (0.00–0.00) **	0.00 (0.00–0.00) **
*Bifidobacterium*	3.34 (2.14–6.20)	0.06 (0.00–6.36) **	0.05 (0.00–0.10) **
*Anaerostipes*	2.93 (2.28–4.75)	0.00 (0.00–0.00) **	0.29 (0.00–1.16) **
*Agathobacter*	2.66 (0.61–7.10)	0.00 (0.00–0.00) **	0.00 (0.00–0.00) **
*Fusicatenibacter*	2.49 (1.56–7.71)	0.00 (0.00–0.00) **	0.00 (0.00–0.00) **
*Eubacterium hallii group*	1.63 (0.36–2.24)	0.00 (0.00–0.07)	0.00 (0.00–0.00)
*Roseburia*	1.62 (1.20–4.98)	0.00 (0.00–0.00) **	0.00 (0.00–0.00) **
*Parabacteroides*	1.59 (1.06–3.19)	9.39 (4.35–19.89) **	0.37 (0.04–3.05) ^†^
*Unclassified*	1.35 (0.65–1.62)	6.76 (5.39–7.48) **	7.89 (3.28–9.12)
*Collinsella*	1.28 (0.45–1.45)	0.00 (0.00–3.27)	0.00 (0.00–0.92)
*Lachnoclostridium*	1.02 (0.63–1.40)	1.32 (0.92–4.77)	2.97 (2.21–3.55)
*Lachnospiraceae NK4A136 group*	0.99 (0.02–1.44)	0.00 (0.00–0.00)	0.00 (0.00–0.00)
*Ruminococcus 2*	0.84 (0.00–4.07)	0.00 (0.00–0.00)	0.00 (0.00–0.00)
*Ruminococcus torques group*	0.74 (0.02–1.56)	0.55 (0.44–0.69)	0.40 (0.00–2.75)
*Alistipes*	0.55 (0.19–0.93)	2.62 (0.94–3.30)	0.48 (0.41–1.60)
*Subdoligranulum*	0.45 (0.03–3.99)	0.00 (0.00–0.00)	0.00 (0.00–0.00)
*Ruminococcus gnavus group*	0.37 (0.06–1.41)	0.71 (0.00–3.12)	1.35 (0.00–10.30)
*Ruminococcaceae UCG-002*	0.13 (0.00–1.96)	0.00 (0.00–0.00)	0.00 (0.00–0.40)
*Sutterella*	0.00 (0.00–0.86)	1.31 (0.00–1.44)	1.23 (0.00–3.84)
*Clostridium innocuum group*	0.00 (0.00–0.03)	2.71 (0.06–3.32)	0.10 (0.02–0.89)

The value indicates the median (IQR) occupancy of total intestinal microbiota. Intestinal bacteria with an occupancy rate ≥1% are shown. ** significant difference (*p* < 0.01) compared with HT. ^†^ significant difference (*p* < 0.05) compared with EN.

**Table 2:** Metabolomic analysis of intestinal metabolites (nmol/g).

Intestinal Metabolites (nmol/g)	HT	EN	EN Pro (+)
Acetic acid	252,580.13 (180,372.79–573,905.12)	129,897.19(112,520.47–209,572.18)	447,528.97 (250,086.40–611437.28)
Propionic acid	47,302.18 (35,579.74–65,092.26)	29,917.86 (27,741.12–37,093.41)	95,408.42 (19,889.65–103,542.40)
Pyruvic acid	1134.66 (481.72–1501.72)	0.00 (0.00–0.00) **	496.94 (296.98–946.55) ^†^
Isobutyric acid	2707.42 (1519.09–4458.13)	4050.38 (3755.59–4878.96)	6012.13 (5238.17–14,997.66)
Butyric acid	29,652.65 (15,291.35–44,766.02)	2924.76 (521.84–6709.13) **	19,645.04 (15,059.10–50,424.18) ^†^
Lactic acid	239.79 (64.23–752.95)	0.00 (0.00–906.65)	154.32 (44.14–265.63)
Isovaleric acid	1753.43 (580.36–3396.05)	3343.35 (2626.70–6838.71)	5457.41 (5423.17–11,377.23)
Valeric acid	3168.25 (2565.22–4186.83)	2009.47 (572.39–3964.96)	3272.69 (2074.14–12,968.91)
2-Hydroxyisobutyric acid	2.29 (0.00–8.51)	33.08 (30.63–60.85) **	0.00 (0.00–42.20)
2-Methylvaleric acid	0.00 (0.00–0.00)	0.00 (0.00–0.00)	0.00 (0.00–0.00)
4-Methylvaleric acid	0.00 (0.00–0.00)	0.00 (0.00–0.00)	0.00 (0.00–0.00)
Caproic acid	0.00 (0.00–825.07)	0.00 (0.00–715.71)	959.72 (614.58–969.63)
Malonic acid	0.00 (0.00–201.50)	0.00 (0.00–370.86)	1041.24 (216.11–1440.59)
Succinic acid	717.54 (627.78–1113.96)	6424.71 (707.86–38,814.07)	6565.67 (5106.95–137,974.49)
LCA	172.05 (113.95–278.99)	211.27 (142.34–973.96)	493.75 (205.04–832.45)
UDCA	6.48 (0.36–50.22)	1.08 (0.57–12.68)	1.42 (1.36–76.44)
CDCA	9.13 (0.47–70.97)	8.04 (6.47–9.57)	1.64 (0.38–37.44)
DCA	201.08 (82.73–267.01)	119.56 (29.05–146.37)	550.86 (499.64–585.78)
CA	5.65 (1.11–41.95)	1.37 (1.30–1.88)	5.88 (1.78–14.70)
GUDCA	0.15 (0.04–0.91)	0.01 (0.01–0.07)	0.06 (0.01–1.39)
GCDCA	1.91 (0.85–8.01)	0.05 (0.04–0.49)	2.11 (2.04–8.37)
GDCA	0.89 (0.50–1.64)	0,05 (0.04–0.33)	1.12 (0.33–2.18)
GCA	1.59 (0.55–4.99)	0.04 (0.03–0.44)	0.95 (0.31–38.20)
TLCA	0.02 (0.00–0.14)	0.02 (0.02–0.03)	0.02 (0.01–0.11)
TUDCA	0.08 (0.01–0.33)	0.01 (0.00–0.01)	0.01 (0.01–0.13)
TCDCA	3.81 (0.27–10.73)	0.02 (0.01–0.06) **	2.61 (0.99–3.02) ^††^
TDCA	0.96 (0.13–4.79)	0.49 (0.05–0.54)	0.71 (0.64–0.91)
TCA	1.32 (0.19–3.86)	0.18 (0.06–0.75)	1.56 (0.18–6.30)

** significant difference (*p* < 0.01) compared with HT; ^†^ significant difference (*p* < 0.05) compared with EN; ^††^ significant difference (*p* < 0.01) compared with EN.

## Data Availability

Data is contained within the article and supplementary materials.

## Supplementary Materials

The following supporting information can be downloaded at: https://www.mdpi.com/article/10.3390/foods11040549/s1, Figure S1: Heat map of intestinal microbiota; Table S1: Correlation analysis between intestinal microbiota and metabolites.
