# Supplementary material for: The Effects of Enteral Nutrition on the Intestinal Environment in Patients in a Persistent Vegetative State
Source: Foods. 2022 Feb 15;11(4):549. doi: 10.3390/foods11040549 (PMC8871387; doi:10.3390/foods11040549)
Supplement: Supplementary file 1 [file foods-11-00549-s001.zip › foods-1571999-supplementary.pdf]

Supplementary Materials

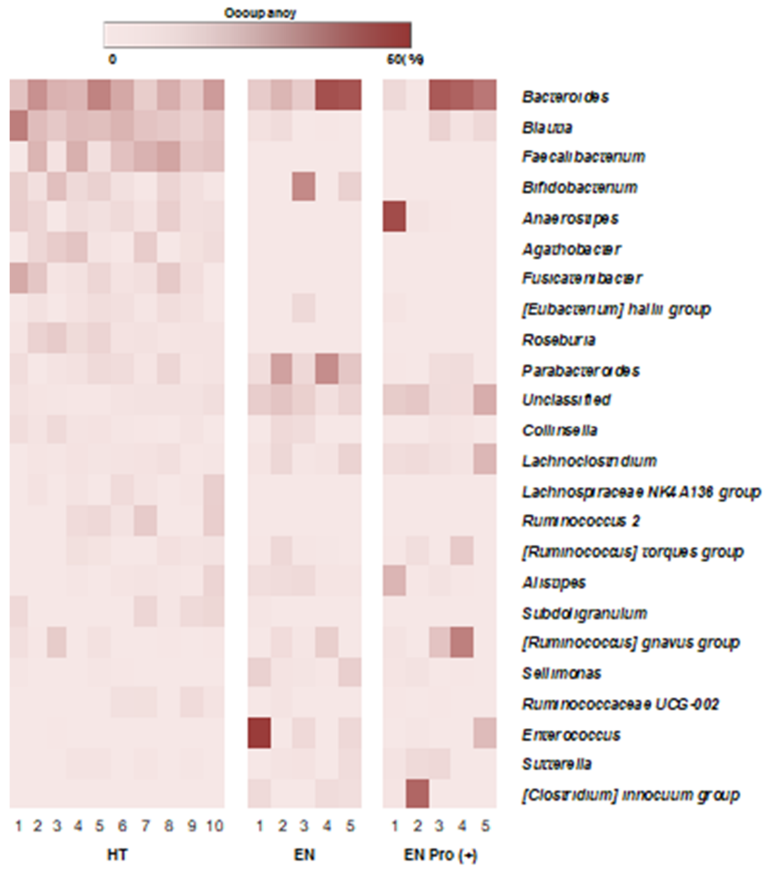

Figure S1. Heat map of intestinal microbiota.

Table S1. Correlation analysis between intestinal microbiota and metabolites.

Metabolites and Intestinal Bacteria	Rs	*p*-Value
Butyric acid		
Blautia	0.467	0.038
Anaerostipes	0.525	0.017
Roseburia	0.496	0.026
Agathobacter	0.520	0.019
Lachnospiraceae NK4A136 group	0.511	0.021
Parabacteroides	-0.547	0.013
Sellimonas	-0.475	0.034
Pyruvic acid		
Anaerostipes	0.724	0.000
Roseburia	0.446	0.049
Fusicatenibacter	0.587	0.007
2-Hydroxyisobutyric acid		
Unclassified	0.485	0.030
Sellimonas	0.665	0.001
(*Clostridium*) *innocuum* group	0.528	0.017

Blautia	−0.490	0.028
Roseburia	−0.491	0.028
Faecalibacterium	−0.551	0.012
Lachnospiraceae NK4A136 group	−0.477	0.033
Alistipes	−0.586	0.007
